# Viscoelastic fluid flow over a horizontal flat plate with various boundary slip conditions and suction effects

**DOI:** 10.1039/d3na00735a

**Published:** 2023-10-17

**Authors:** K. Sudarmozhi, D. Iranian, M. Asif Memon, P. D. Selvi, M. Sabeel Khan, Amsalu Fenta

**Affiliations:** a Department of Mathematics, Saveetha School of Engineering, SIMATS Chennai Tamil Nadu India sudarmozhik1033.sse@saveetha.com Iranian.sse@saveetha.com; b Department of Mathematics and Social Sciences, Sukkur IBA University Sukkur 65200 Sindh Pakistan asif-memon@iba-suk.edu.pk; c Academic Consultant, Department of Applied Mathematics, Sri Padmavathi Mahila Visva Vidyalayam Tirupati India selvi.pd2014@gmail.com; d Department of Mathematics, Capital University of Science and Technology 44000 Islamabad Pakistan muhammad.sabeel@cust.edu.pk; e Department of Physics, Mizan Tepi University PO Box 121 Tepi Ethiopia fentaamsalu0923@gmail.com

## Abstract

This study examines the numerical representation of fluid flow on the Maxwell model in a double-diffusive boundary layer over a horizontal plate. The investigation incorporates slip conditions, encompassing momentum slip, thermal slip, and suction parameters. Moreover, the study includes the inspiration of thermal radiation, heat generation, and mass transfer. The governing partial differential equations (pertaining to momentum, continuity, energy transport, and mass transport) are transformed into ordinary differential equations (ODEs) using appropriate similarity transformations. To solve these equations in conjunction with suitable boundary conditions, the bvp4c inbuilt software is implemented. This is achieved through the shooting approach employed in MATLAB. A comprehensive agreement between the numerical technique and previously published findings demonstrates its efficacy. The outcomes are presented through graphical representations and tables, showcasing various parameters such as momentum slip, temperature slip, local Nusselt number, Sherwood number, and suction parameter. The primary motivation of this research lies in investigating the behaviour of Maxwell fluid flow in the absence of slip conditions. The study of Maxwell fluid flow over a flat plate with the combined effects of suction, thermal slip, and momentum slip conditions has a wide range of practical applications that span multiple industries, contributing to improved designs, efficiency, and understanding of fluid behaviour in various systems. The main aim of this study is to present streamlined results under varying conditions, explicitly investigating the influence of suction effects and slip conditions on the flow.

## Abstract 2

This study examines the numerical representation of fluid flow on the Maxwell model in a double-diffusive boundary layer over a horizontal plate.

## Graphical abstract

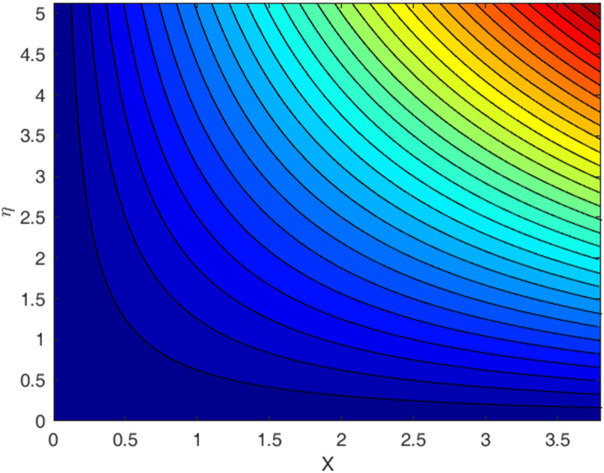

## 1 Introduction

Due to the importance of heat transmission in the design and operation of heating and cooling systems, thermal engineering strongly depends on the study of heat transfer. In several scientific domains, heat transfer and solute transport play an important role, including nuclear reactors, plastic extrusion, electronic appliance cooling, and polymer manufacturing. The study of suction, thermal slip, and momentum slip in the context of Maxwell fluid flow over a horizontal plate is of great importance for understanding the behaviour of complex viscoelastic fluids in practical applications. These effects influence the stress distribution, temperature profile, and velocity profile near the boundary, and their consideration is crucial for optimizing various engineering processes involving Maxwell fluid. Researchers and engineers continue to explore these effects to develop more accurate models and design guidelines for applications involving these unique fluids.

Following this, non-Newtonian fluids find applications in areas such as decreasing friction, notably in contexts such as oil pipelines. Devi *et al.*^1^ made several noteworthy discoveries in transport theory. In a numerical study, Venkatadri *et al.*^2^ discussed the MHD radiative heat transfer investigation of Carreau nanofluid flow past a perpendicular plate. Madhavi *et al.*^3^ analyzed the entropy analysis of MHD convection flow from a flat cylinder with slip. Ramesh Reddy *et al.*^4^ studied the entropy of mixed convection flows of tangent hyperbolic fluid across an isothermal wedge. Beg and Makinde^5^ researched the Maxwell fluid flow and mass transfer in a Darcian porous channel. Numerous researchers have explored diverse categories of non-Newtonian fluids and various geometries of Maxwell fluids. Ahmed^6^ described Maxwell fluid on a stretching sheet. The consequence of radiation over Maxwell fluid on a porous sheet was explored by Mukhopadhyay *et al.*^7^ Yu Bai *et al.* provided reports on the Maxwell nanofluid flow.^8^

Radiative fluid flow is an essential part of the design of propellers, rockets, satellites, artillery, fissionable power plants, and other multifunctional reduction structures. Heat generation on a flat plate is fundamental to various engineering systems and processes. Its control and management are critical for these systems' efficient and safe operation. Engineers and researchers continually explore innovative methods to optimize heat generation and its effects, leading to technological advancements, energy efficiency, and industrial processes. The outcome of thermal radiation on a porous surface heated by convection was calculated by Hosseinzadeh *et al.*^9^ They proposed that there is a one-to-one correspondence between the temperature distribution and the profile of radiation. In the occurrence of radiation, Rashid *et al.*^10^ described a Maxwell fluid spinning over a porous three-dimensional surface. Samuel *et al.*^11^ considered energy transfer in Maxwellian radiation flow across an exothermic surface and considered the possessions of thermal conductivity & variable viscosity. Maxwell fluid flow with radiation across a surface of varying thickness was solved numerically by Elbashbeshy *et al.*^12^ Non-Newtonian Maxwell fluid flow along a linear sheet was discussed by Khan *et al.*^13^ Hayat *et al.*^14^ calculated thermophoresis and heat radiation effects using magneto-hydrodynamic Maxwell fluid on a linear sheet. Hayat *et al.*^15^ presented a non-Newtonian Maxwell fluid flow with thermal radiation's impact in a porous media-filled environment. In ref. 16, Mahanthesh *et al.* investigated a new 3D flow of a nonlinear radiative transfer equation. Numerous sectors of industry and technology can benefit from the findings of numerical research, including momentum slipping conditions and the thermal transfer mechanism. Reddy *et al.*^17^ examined the study of the magnetohydrodynamics (MHD) boundary layer slip flow of a Maxwell nanofluid across an exponentially stretching surface with convective boundary conditions. Ibrahim and Negera^18^ analyzed the MHD slip flow of upper-convected Maxwell nanofluid across a sheet with a chemical reaction. Gowda *et al.*^19^ examined the slip flow of Casson–Maxwell nanofluid confined over stretchable disks. Ghalib *et al.*^20^ examined the non-time-dependent MHD flow of Maxwell fluid with slip/non-slip fluid flow and Newtonian heating at the boundary. Shamshuddin *et al.*^21^ researched the bioconvection nanofluid flow. Dogonchi *et al.*^22^ analyzed the thermal and entropy investigations on buoyancy-driven nanofluid flow inside a porous enclosure with two square cylinders. Shamshuddin *et al.*^23^ showed a numerical analysis focusing on heat transfer and viscous flow within a dual-rotating extendable disk system. Pattnaik *et al.*^24^ explored the influence of varying the shape of Fe_3_O_4_-nanoparticles on energy transfer phenomena, incorporating thermal radiation in their study. In the work of Pattnaik *et al.*,^25^ a numerical simulation was carried out to observe the flow characteristics of conducting metal and metallic oxide nanofluids. Pattnaik *et al.*^26^ delved into a comprehensive study involving mixed convective-radiative dissipative magnetized micropolar nanofluid flow on a stretching surface in permeable media, considering double stratification and chemical reaction impacts. Mohanty *et al.*^27^ employed numerical methods to analyze magnetohydrodynamic (MHD) nanofluid flow on a stretching surface, taking into account radiation. Mishra *et al.*^28^ inspected the impact of radiation and cross-diffusion influence on micropolar nano-liquid flow over a stretching sheet featuring a heat source. Damseh *et al.*^29^ discussed the effects of heat generation and first-order chemical reactions in micropolar fluid flows on a uniformly stretched porous surface. Krishna *et al.*^30^ delved into the effects of Joule, Soret, and Hall impacts on MHD rotating mixed convective flow past an infinite perpendicular permeable plate. Chamkha and Khaled^31^ examined solutions for hydromagnetic, energy and mass transfer in the context of free convection from an inclined plate, considering internal energy generation. Rama SubbaReddy Gorla and Ali Chamkha^32^ investigated the free convective boundary layer flow on a non-isothermal perpendicular plate embedded in a porous medium. Kumar *et al.*^33^ studied the influence of an induced magnetic field and radiation on the magneto-convection flow of a dissipative fluid. Takhar *et al.*^34^ analysed MHD flow on a moving plate in a rotating fluid, considering the influence of a magnetic field.

### 1.1 Objective of this study

Examining the flow of Maxwell fluid over a flat plate, while considering the combined influence of thermal slip and momentum slip conditions, along with the effect of suction, constitutes a research endeavour situated within the domains of fluid dynamics and heat transfer. This study has implications for industries such as polymer processing and materials manufacturing. Including slip conditions (momentum and thermal slip) reflects real-world scenarios where fluid-solid interactions are not purely no-slip. Studying these slip conditions is essential for applications such as microfluidics, where slip effects become significant due to the small scale of the system. The introduction of suction at the plate's surface is often used to control or manipulate the flow in various engineering applications. Understanding the impact of these boundary conditions on Maxwell fluid flow can help design systems with enhanced control over fluid behaviour.

### 1.2 The novelty of this research

According to the literature, the thermal radiation and momentum slip influence of the boundary layer over a Maxwell fluid flow on a horizontal plate in terms of heat mass transfer across a flat plate has yet to be thoroughly investigated by any other authors in the literature. All others in the literature researched only Maxwell fluid flow on a horizontal scale with the combination of fewer parameters, and few more authors have performed their work in different fluids and geometries. So, the critical goal of this study is to solve governing equations involving radiation, heat generation, velocity slip conditions, thermal slip conditions, and suction parameter. A system of ODEs was solved in MATLAB-encoded bvp4c after the similarity transformation was carried out successfully for PDEs. Validation is achieved through comparison to a current study. The results are given for streamlining the suction effect and slip conditions for different values.

## 2 Mathematical modeling

This article developed the theory of Maxwell fluid flow in a horizontal plate's boundary layer at a steady state. The u* & v* velocity mechanisms are measured along the *x* & *y* axes. Heat generation, radiation, velocity slip, and suction effect are measured. The elongation of the flat plate occurs along the *x*-axis. Temperature *T* and solute concentration *C* are held constant at *T*_w_ and *C*_w_ along the *y*-axis of the horizontal plate (*i.e.*, *y* = 0). The ambient concentration is denoted as *C*_∞_, respectively, at *y* > 0. Using the Oberbeck–Boussinesq approximation, we examine the incompressible, laminar flow of the Maxwell boundary layer across a flat plate. *q*_r_ is the radiative heat flux calculated using *q*_r_ = −(4σ*/3k*)∂*T*^4^/∂*y*. Here, σ*and k* represent the Stephan–Boltzmann coefficient and the mean absorption coefficient. Haughty that the temperature variances in the flow are small enough, *T*^4^ can be represented as a linear function of temperature. This is achieved by mounting *T*^4^ about the ambient temperature *T*_∞_ and ignoring the higher-order terms to arrive at *T*^4^ ≅ 4*TT*_∞_^3^ − 3*T*_∞_^4^ at the solution.

### 2.1 Applications related to this problem

Studying Maxwell fluid flow on a horizontal plate with the combined effects of suction, radiation, thermal slip, and velocity slip conditions has applications in several fields, particularly in advanced engineering, materials processing, and aerospace.

• In this scenario, studying Maxwell fluid flow with the combined impacts of suction, radiation, thermal slip, and velocity slip conditions allows engineers and researchers to optimize the extrusion process for improved film quality, reduced energy consumption, and increased production rates.

• Investigating the combined effect of thermal slip and radiation to ensure that the polymer melt solidifies at the desired rate and temperature, preventing defects such as wrinkles and bubbles in the film.

• Understanding how momentum and thermal slip conditions affect the velocity and temperature profiles near the flat plate can help design optimal cooling and suction systems.

• Optimizing suction and radiation can lead to energy-efficient processes, reducing costs and environmental impacts.

• A better understanding of the complex fluid dynamics and energy transfer in the extrusion process can improve film quality, reduce waste, and enhance product performance.

### 2.2 Equation of continuity in the dimensional form

The continuity equation is often combined with other equations, such as the Navier–Stokes equations for fluid motion, to analyze and solve complex fluid flow problems. It provides valuable insights into the behavior of fluids and is a fundamental tool in engineering and physics (from ref. 35 and 36).1
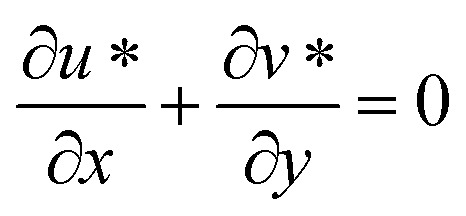


### 2.3 Momentum conservation in the dimensional form



2

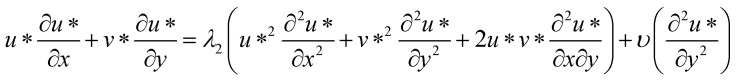

In a Maxwell fluid flow, the behavior of the fluid can be described by a differential equation known as the Maxwell model, which relates the shear stress (*τ*) to the rate of change of shear rate (d*u*/d*t*) and the relaxation time (*λ*_2_) of the fluid.

### 2.4 Energy conservation in the dimensional form [using ref. 37–41]



3

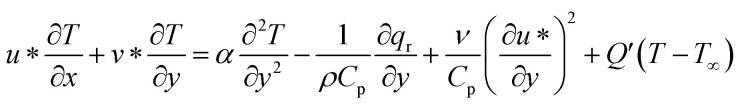

Heat generation on a flat plate can profoundly affect the performance and behavior of various systems. It is a critical factor in applications across multiple industries, including electronics cooling, aerospace and automotive engineering, industrial processes, and energy generation.

### 2.5 Mass equation in dimensional form



4

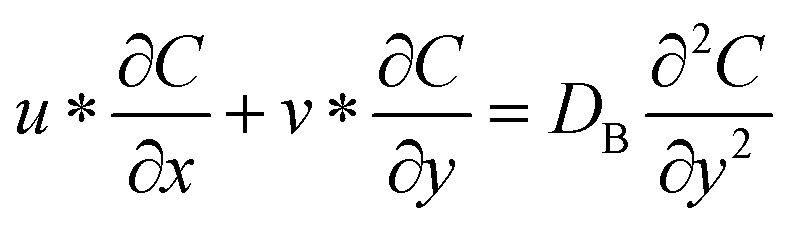

Mass transfer over a horizontal plate is a fundamental chemical engineering and fluid dynamics phenomenon. It plays a crucial role in various industrial processes and applications.

### 2.6 Boundary conditions of the flat plate

Momentum slip occurs when there is a difference in the velocity between the fluid and the solid boundary. In Maxwell fluid flow over a horizontal plate, the momentum slip effect can modify the velocity profile near the surface, leading to changes in the boundary layer thickness and shear stress distribution. This has significant implications for drag reduction and control of fluid flow. Thermal slip refers to the difference in temperature at the fluid-solid interface, leading to heat transfer effects that affect the boundary layer development [from ref. 42].5
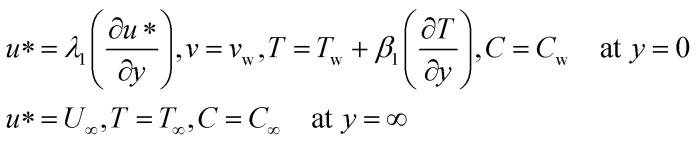
Here, the thermal slip length (*β*_1_) characterizes how the fluid temperature deviates from the temperature of the solid surface as one moves away from the boundary. A more considerable thermal slip length indicates a more significant departure from the solid surface temperature. In contrast, a smaller thermal slip length implies that the fluid temperature closely matches the solid surface temperature. As with the velocity slip length, the thermal slip length typically represents the distance over which the temperature gradient in the fluid influences the heat transfer behavior near the solid boundary. The suction of fluid at the boundary has a significant influence on the flow dynamics. Suction denotes the removal of fluid from the boundary. In the context of Maxwell fluids, the occurrence of suction can alter the stress distribution near the flat plate.

Let us introduce dimensionless quantities to convert PDEs into ordinary differential equations (from ref. 43).6
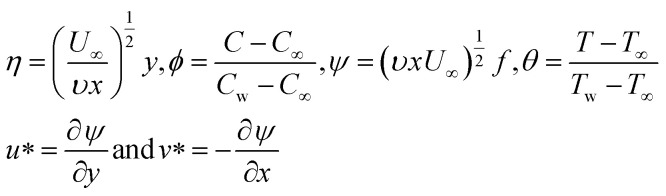


• 
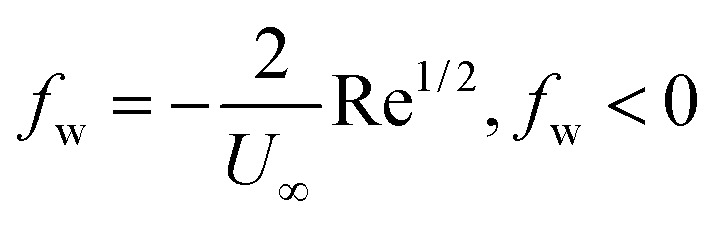
 represents injection (*v*_w_ > 0) and *f*_w_ > 0 represents suction (*v*_w_ < 0).

• 
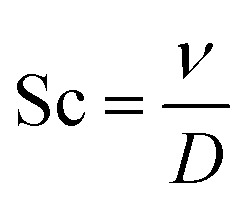
 represents the Schmith number.

• 
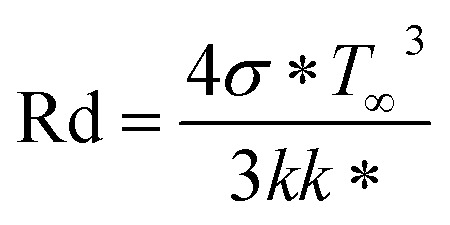
 represents radiation.

• 
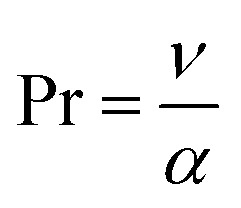
 represents the Prandtl number.

• 
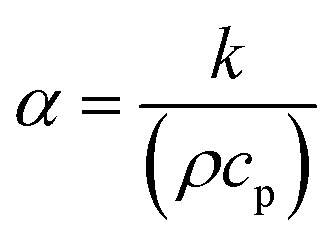
 represents thermal diffusivity.

• 
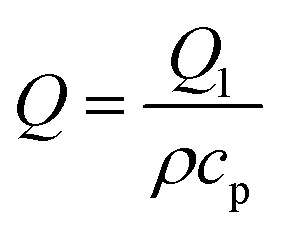
 represents heat generation.

• 
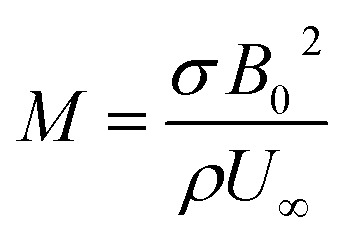
represents the magnetic field.

• 
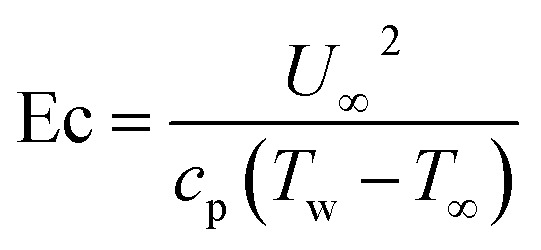
 represents the Eckart number.

• 
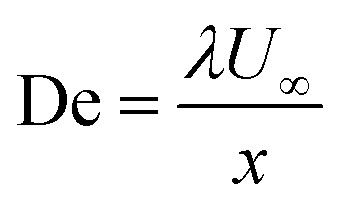
 represents the Deborah number.

• Velocity slip is a phenomenon that occurs when a fluid flows on a solid surface, such as a flat plate, and the fluid velocity at the surface is different from the velocity of the solid surface itself. This effect contrasts with the “no-slip” boundary conditions, which assume that the fluid velocity at the solid surface is zero and the fluid adheres to the surface. In the case of a horizontal plate, if there is a velocity slip effect, the fluid particles near the solid surface do not adhere to the surface and have a non-zero velocity component tangential to the surface. This slip velocity is typically denoted as “*λ*” and represents the relative motion between the fluid and the solid surface. Various factors, including the nature of the surface, surface roughness, and the presence of a thin layer of gas or other materials near the surface can cause the slip velocity.

• Thermal slip, similar to velocity slip, is a phenomenon that occurs when a fluid flows on a solid surface, such as a flat plate, and the temperature of the fluid at the surface differs from the temperature of the solid surface itself. This effect is in contrast to the “no-slip” thermal boundary conditions, which assume that the fluid temperature at the solid surface is equal to the temperature of the surface. In the case of a flat plate with a thermal slip effect, it means that the fluid near the solid surface does not instantly adjust to the temperature of the solid, and there is a temperature difference between the fluid and the solid. This thermal slip is typically denoted as “*β*” and represents the difference in temperature between the fluid and the solid surface at the point of contact. The thermal slip effect can be caused by various factors, including the solid surface's thermal properties, a thin insulating layer, and the nature of the fluid-solid interaction.

PDEs often involve numerous physical parameters, such as viscosity, density, velocity, and length scales. Non-depersonalization helps simplify these equations by expressing these parameters in terms of dimensionless numbers. This decreases the number of parameters and makes the equations more manageable. Using dimensionless quantities and similarity variables, the PDEs turned into ODEs. ODEs are generally easier to solve numerically than PDEs. Non-dimensional PDEs can simplify the numerical solution process, reducing the computational effort and resources required for simulations. The use of non-dimensional quantities in converting PDEs to ODEs is a valuable technique for simplifying complex problems, making them more tractable, and providing valuable insights into the behaviour of physical systems.

### 2.7 Momentum conservation in the dimensionless form

Maxwell fluids find applications in various fields, including rheology, polymer science, and modeling certain types of complex fluids, such as certain suspensions and emulsions. Understanding the behavior of Maxwell fluids is essential for designing and analyzing systems involving these materials.7
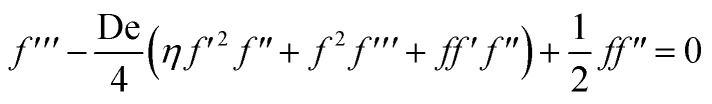


### 2.8 Energy conservation in the dimensionless form



8

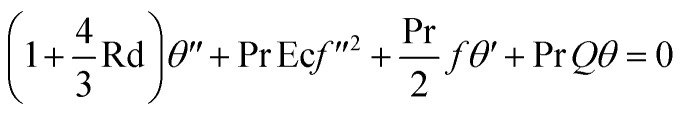




### 2.9 Mass equation in dimensionless form



9

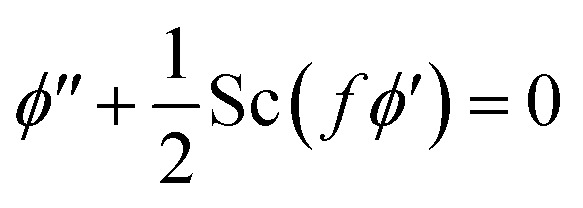




### 2.10 The appropriate equivalent dimensionless boundary conditions are given here



10

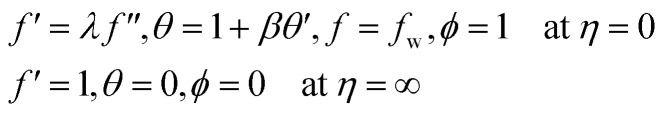

The dimensionless suction parameter describes the fluid's mass flow rate at a solid surface. Slip conditions, on the other hand, pertain to the behavior of fluid near the solid–fluid interface, where the slip length characterizes the degree of slippage. Both concepts are essential in various fluid dynamics and heat transfer applications.

The Nusselt and Sherwood numbers are crucial in analyzing and designing systems involving heat transfer and mass transfer, respectively. They provide insights into the efficiency of these processes and are used to develop practical engineering solutions in various industries, including aerospace, chemical engineering, thermal management, and environmental engineering.

By engaging the Fourier and Fick's laws, Cf, Nu_*x*_ & Sh_*x*_ quantities are defined below.11
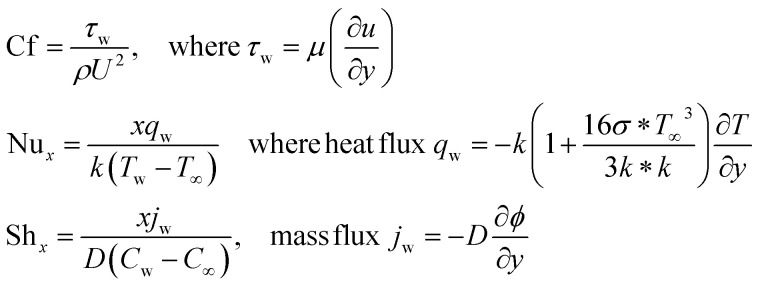


## 3 Numerical method and its validation

This section of the report presents the numerical solution addressing the research problem. Due to the pronounced nonlinearity inherent in the problem, our numerical approach revolves around solving a set of interconnected ODEs that come with intricate boundary conditions. Dealing with these highly nonlinear boundary values represents a substantial analytical challenge that should not be underestimated. To overcome this hurdle and solve boundary value problems (BVPs) entailing multiple boundary conditions for ordinary differential equations, we employ BVP4C, a specialized solver meticulously designed for this precise purpose. The choice between these methods depends on the nature of the differential equation problem, whether it involves boundary or initial value problems. BVP4C is a numerical method used to solve boundary value problems associated with ODEs. It is particularly useful when analytical solutions are not readily available or when dealing with complex systems in science and engineering. Researchers and engineers often use software packages such as MATLAB to implement BVP4C and obtain numerical solutions to their BVPs.

The substitutions are as follows:
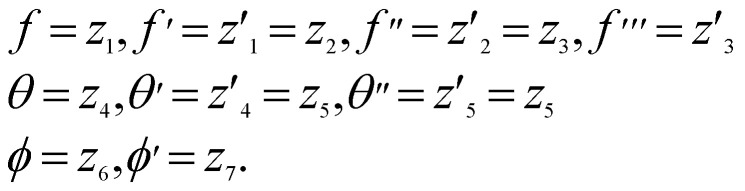

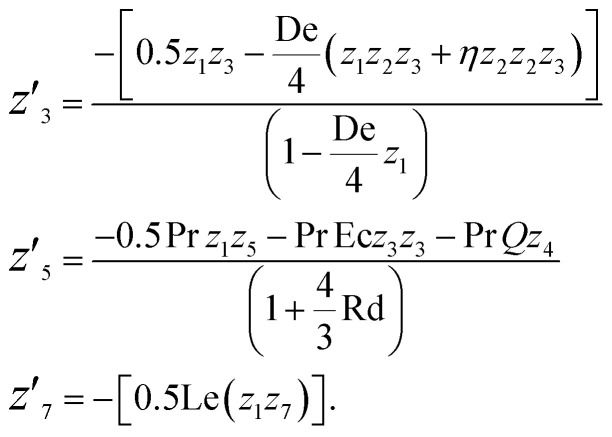


### 3.1 Algorithm for the proposed method

Step 1: Define the boundary value problem: define the problem by defining the differential equation(s), boundary conditions, and additional parameters.

Step 2: Initialize guess for solution: start with an initial guess for the solution to the boundary value problem.

Step 3: Numerical integration loop: this loop iteratively refines the solution until it converges to the desired accuracy.

Step 4:

(a) Solve the ODE: use a numerical method such as bvp4c in MATLAB to solve the ordinary differential equations associated with the BVP.

(b) Check convergence: check whether the solution has converged to the desired accuracy. If yes, exit the loop. If not, proceed to the next step.

(c) If not converged, update guess: adjust the initial guess for the solution based on the current solution and repeat the integration loop.

Step 5: Output solution: once the solution has converged, output the final solution to the boundary value problem.
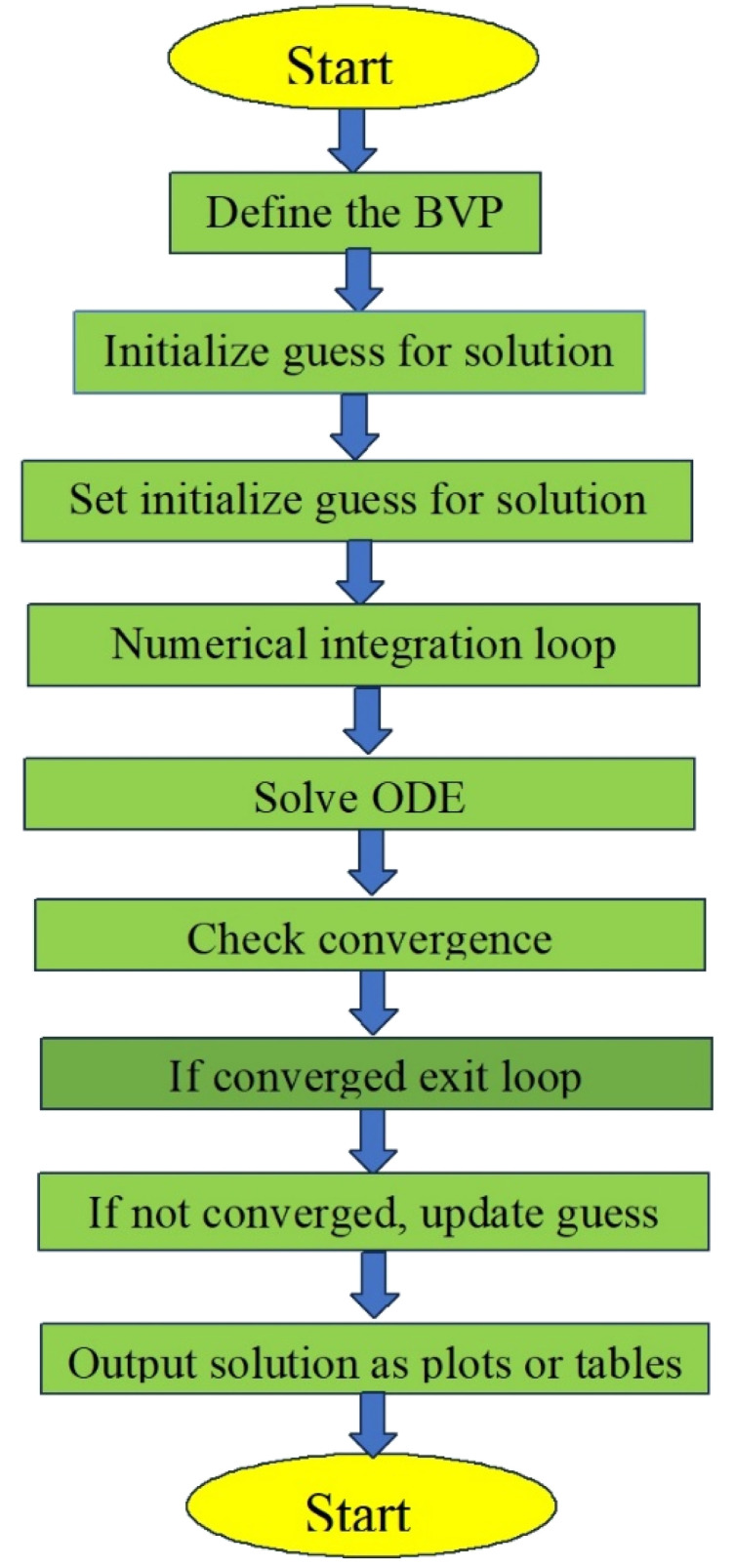


### 3.2 Validation of the problem

Table 1 illustrates a remarkable resemblance between our research findings and those of Hayat *et al.*^44^ After confirming our numerical method's validity, we will present the computational results for the Maxwell model applied to a flat plate.

**Table 1:** Validation of *C*_f_ for different values of *K*, *M* and *f*_w_

*f*_w_	0.0	0.2	0.5	0.7	0.5	0.5	0.5
*M*	1.5	1.5	1.5	1.5	1.0	1.2	1.5
*K*	0.5	0.5	0.5	0.5	0.5	0.5	0.5
Existing work Hayat^44^	1.223676	1.412204	1.646600	1.765886	0.967329	1.278197	1.646600
Present	1.223675	1.141220	1.646599	1.765884	0.967328	1.278196	1.646599

Table 1 represents the validation with existing work for the computation method and we got very good matching values so we have validated our code for this problem.

## 4 Results and discussion

This study examines the numerical representation of fluid on the Maxwell model in a double-diffusive boundary layer on a horizontal plate. The investigation incorporates slip conditions, encompassing momentum slip, thermal slip, and suction parameters. Moreover, the study incorporates the effects of radiation, heat generation, and mass transfer. By utilizing appropriate similarity transformations, the governing PDEs (pertaining to momentum, continuity, energy transport, and mass transport) are transformed into ODEs. To solve this system of equations in conjunction with suitable boundary conditions, we used built-in “bvp4c” software in MATLAB. A comprehensive agreement between the numerical technique and previously published findings demonstrates its efficacy. The outcomes are presented through graphical representations and tables, showcasing parameters such as momentum slip, temperature slip, local Nusselt number, Sherwood number, and suction parameter. Maxwell fluid flow across a horizontal plate is a popular topic of computational research in the scientific literature. Here, we have discussed the theoretical aspects of this issue, including thermal slip, momentum slip, suction, thermal radiation, Eckart number (Ec), and heat generation. Unless otherwise stated, this study's default/unchanged parameters are Pr = 6.8, Ec = 0.1, De = 0.1, Rd = 1, *Q* = 0.1, *λ* = 0.5, *f*_w_ = 1.0, *β* = 0.5, and Sc = 2.0.

Tables 2 and 3 show the computational results of the temperature gradient, Sherwood number and Nusselt number for various physical variables. Table 2 reveals that Nu_*x*_ is boosted with Rd, *Q*, and Ec while it is reduced when the values of *λ*, *β*, and *f*_w_ increase. The Nusselt number Nu_*x*_ is a dimensionless number that plays a crucial role in flat plate boundary value problems, particularly in the context of forced convection heat transfer. The Nusselt number quantifies the convective heat transfer rate from a horizontal plate to a moving fluid. It is used to characterize heat transfer efficiency and is often employed in the analysis and design of heat exchangers, cooling systems, and various other engineering applications.

**Table 2:** Nusselt number values for various parameters with Pr = 6.8, De = 0.1, and Sc = 2.0

*f*_w_	*Q*	Ec	Rd	*β*	*λ*	Nu_*x*_
1.0	0.1	0.1	1	0.5	0.5	0.2117
1.0	0.1	0.1	1	0.5	0	0.2339
1.0	0.1	0.1	1	0.5	0.3	0.2241
1.0	0.1	0.1	1	0.5	0.5	0.2117
1.0	0.1	0.1	1	0.5	1	0.2066
1.0	0	0.1	1	0.5	0.5	0.2060
1.0	0.1	0.1	1	0.5	0.5	0.2117
1.0	0.2	0.1	1	0.5	0.5	0.2223
1.0	0.3	0.1	1	0.5	0.5	0.2268
1.0	0.1	0.1	0	0.5	0.5	0.0628
1.0	0.1	0.1	1	0.5	0.5	0.2117
1.0	0.1	0.1	2	0.5	0.5	0.3701
1.0	0.1	0.1	3	0.5	0.5	0.5344
1.0	0.1	0.1	1	0.3	0.5	0.2395
1.0	0.1	0.1	1	0.5	0.5	0.2117
1.0	0.1	0.1	1	0.9	0.5	0.1599
1.0	0.1	0.1	1	1.2	0.5	0.1346
0	0.1	0.1	1	0.5	0.5	0.2445
0.5	0.1	0.1	1	0.5	0.5	0.1321
1.0	0.1	0.1	1	0.5	0.5	0.2117
1.5	0.1	0.1	1	0.5	0.5	0.1818
1.0	0.1	0	1	0.5	0.5	0.2056
1.0	0.1	0.1	1	0.5	0.5	0.2117
1.0	0.1	0.2	1	0.5	0.5	0.2178
1.0	0.1	0.3	1	0.5	0.5	0.2239

Sh_*x*_ is shown in Table 3 for multiple values. A gradual increment is observed with different values of *λ*, *f*_w_ and Sc. The Sherwood number is dimensionless and provides information about the mass transfer behavior near the flat plate's surface. It depends on factors such as the flow velocity, fluid properties, and the concentration difference between the fluid and the plate surface.

**Table 3:** Sherwood number values for various parameters

*λ*	*f*_w_	Sc	Sh_*x*_
0.5	1.0	2	1.3205
0	1.0	2	1.2223
0.3	1.0	2	1.2890
0.5	1.0	2	1.3205
1	1.0	2	1.3731
0.5	0	2	0.4944
0.5	0.5	2	0.8875
0.5	1.0	2	1.3205
0.5	1.5	2	1.7759
0.5	1.0	2	1.3205
0.5	1.0	3	1.8305
0.5	1.0	5	2.8337
0.5	1.0	9	4.8273

Engineers and scientists use correlations or experimental data to determine the local mass transfer coefficient and calculate the Sherwood number for specific flow conditions. The Sherwood number is critical for predicting the mass transfer rate and concentration distribution in forced convection mass transfer situations involving flat plates. It is applicable in various fields, including chemical engineering, environmental engineering, and food processing, where mass transfer processes play a vital role.

### 4.1 Fluid flow field in the absence of slip conditions

**Fig. 1:**
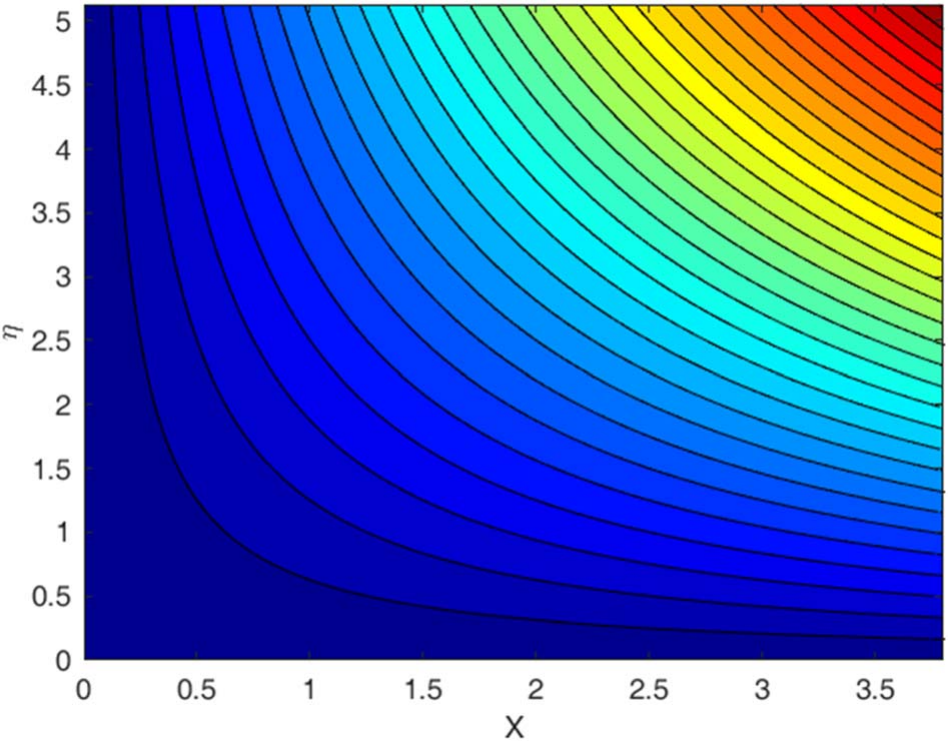
Streamlines for the case *f*_w_ = 0, with Pr = 6.8, Ec = 0.1, De = 0.1, Rd = 1, *Q* = 0.1, *λ* = 0.5, *β* = 0.5, and Sc = 2.0.

**Fig. 2:**
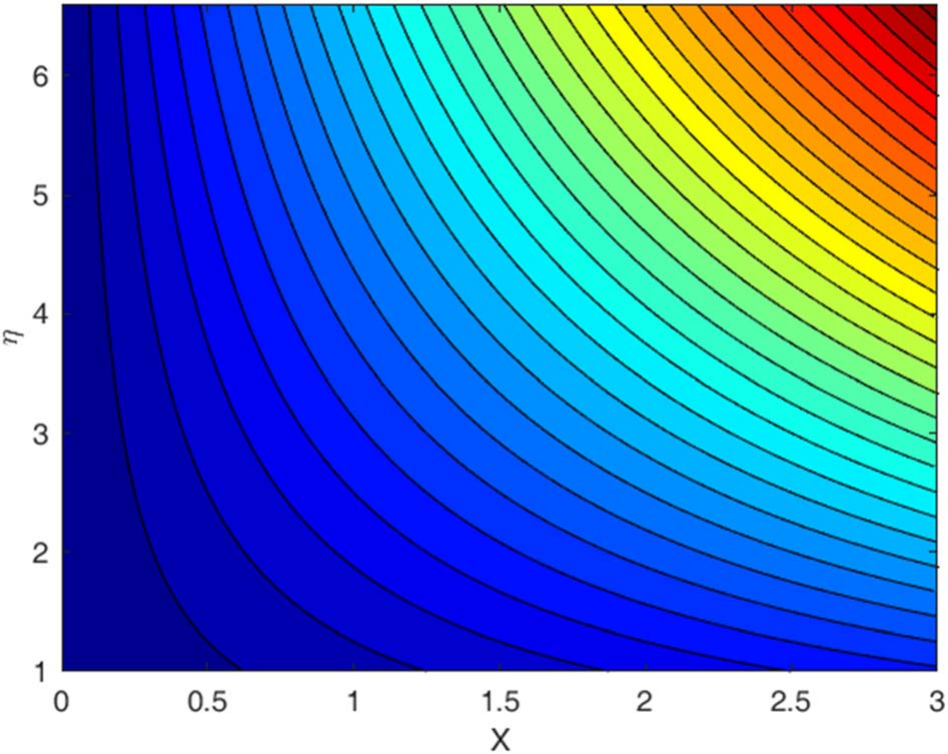
Streamlines for the case *f*_w_ = 1.0, with Pr = 6.8, Ec = 0.1, De = 0.1, Rd = 1, *λ* = 0.5, *Q* = 0.1, *β* = 0.5, and Sc = 2.0.

**Fig. 3:**
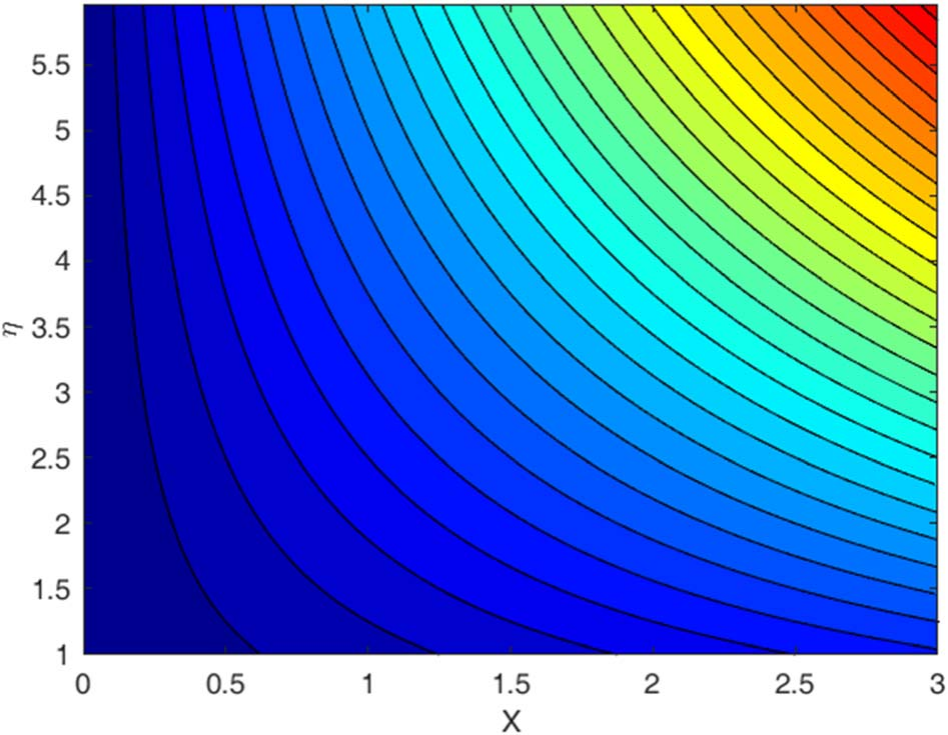
Streamlines for the case *λ* = 0, with *f*_w_ = 1.0, Ec = 0.1, De = 0.1, *Q* = 0.1, Pr = 6.8, Rd = 1, *β* = 0.5, and Sc = 2.0.

**Fig. 4:**
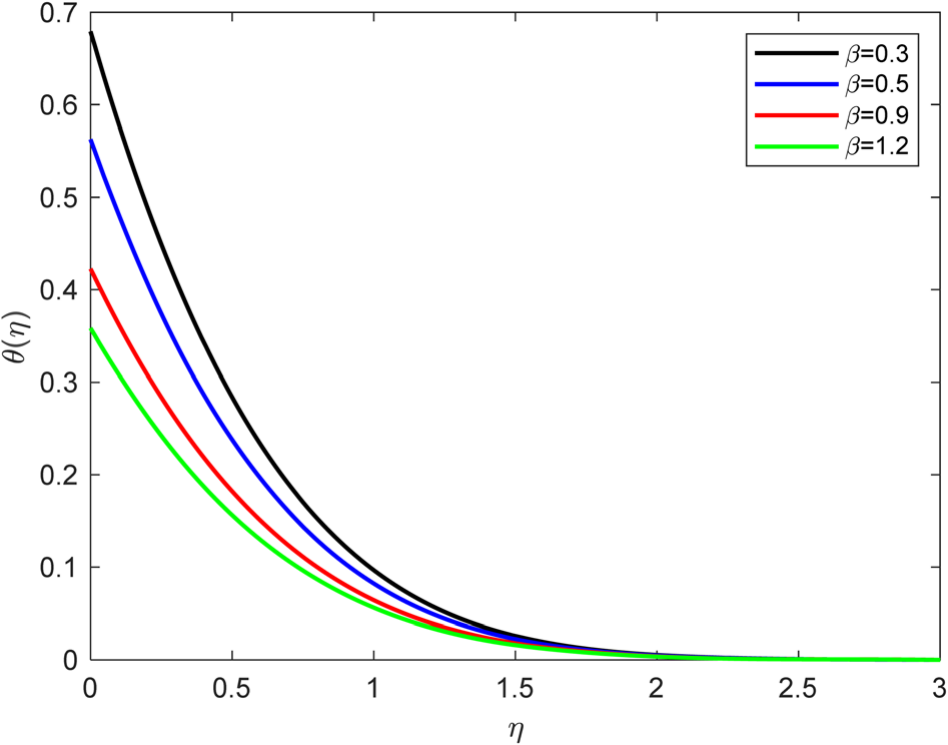
Temperature outline for growth of *β* for Ec = 0.1, De = 0.1, Pr = 6.8, Rd = 1, *Q* = 0.1, *λ* = 0.5, *f*_w_ = 1.0, and Sc = 2.0.

**Fig. 5:**
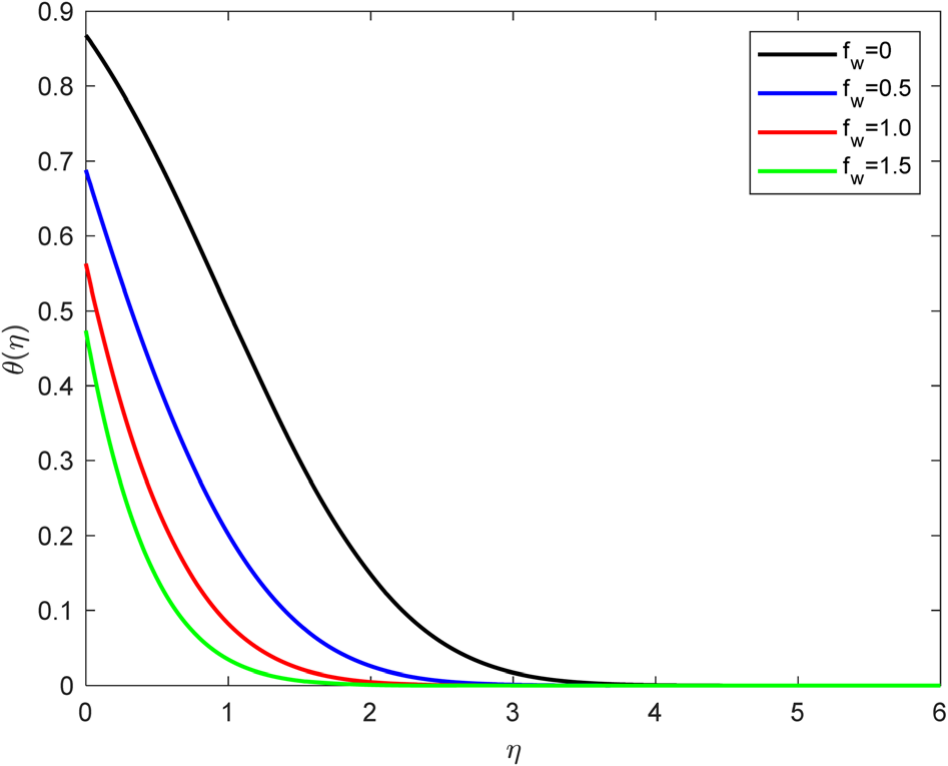
Temperature outline for growth of *f*_w_ for Pr = 6.8, Ec = 0.1, De = 0.1, *Q* = 0.1, *λ* = 0.5, Rd = 1, *β* = 0.5, and Sc = 2.0.

**Fig. 6:**
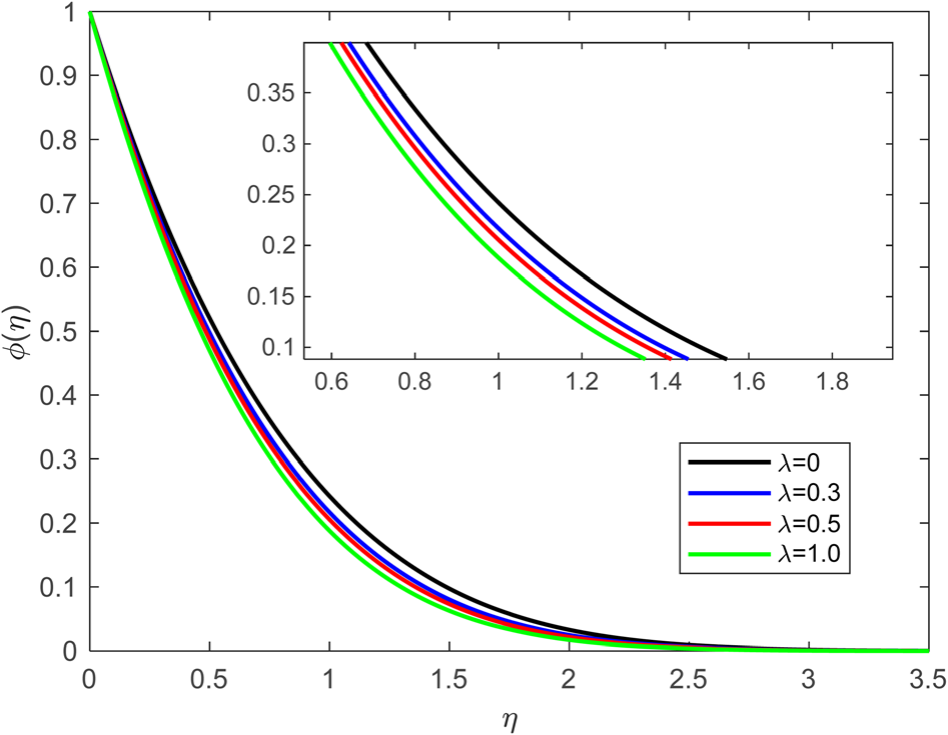
Concentration outline for growth of *λ* for Pr = 6.8, Ec = 0.1, De = 0.1, *Q* = 0.1, *f*_w_ = 1.0, Rd = 1, *β* = 0.5, and Sc = 2.0.

**Fig. 7:**
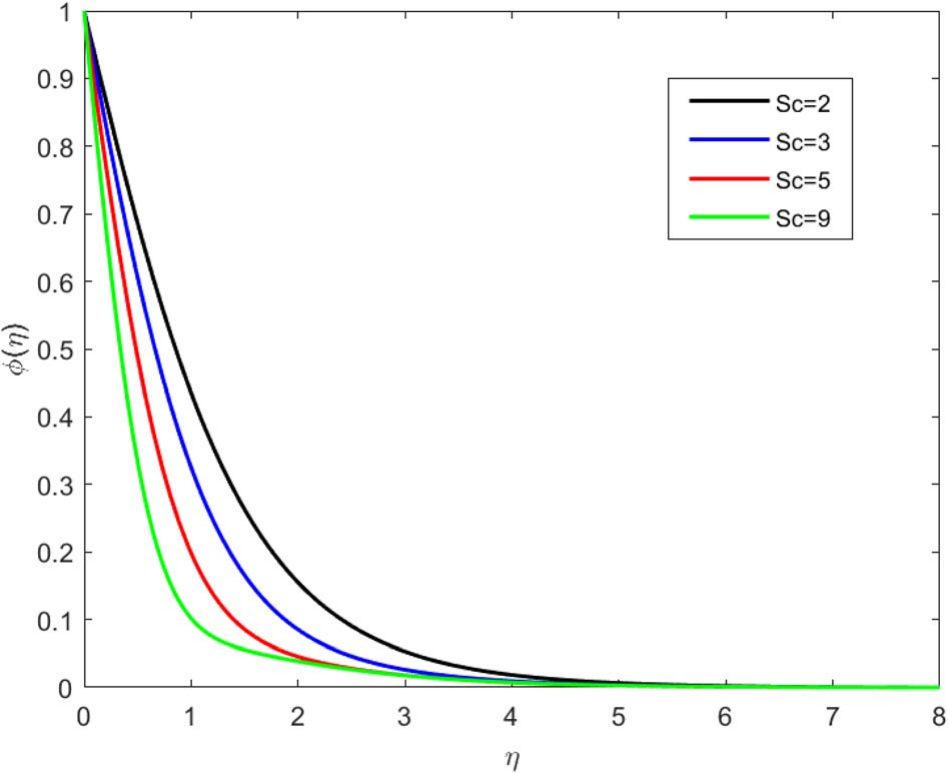
Concentration outline for growth of Sc for Ec = 0.1, De = 0.1, Rd = 1, *Q* = 0.1, Pr = 6.8, *λ* = 0.5, *f*_w_ = 1.0, and *β* = 0.5.

**Fig. 8:**
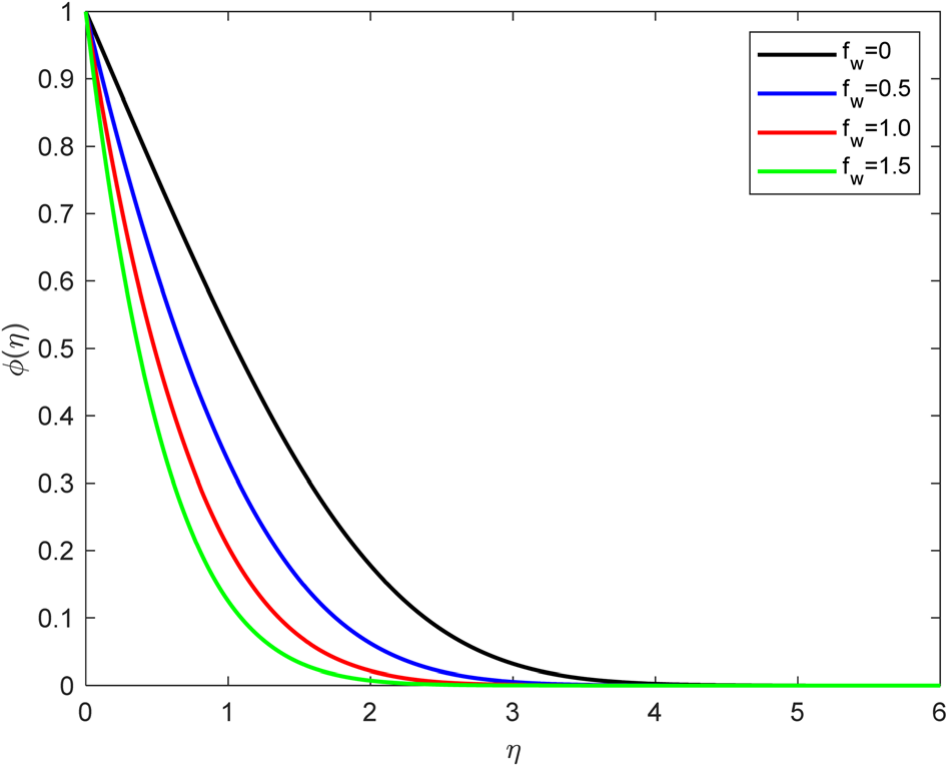
Concentration outline for growth of *f*_w_ for Pr = 6.8, Ec = 0.1, De = 0.1, *Q* = 0.1, *λ* = 0.5, *β* = 0.5, Rd = 1, and Sc = 2.0.

**Fig. 9:**
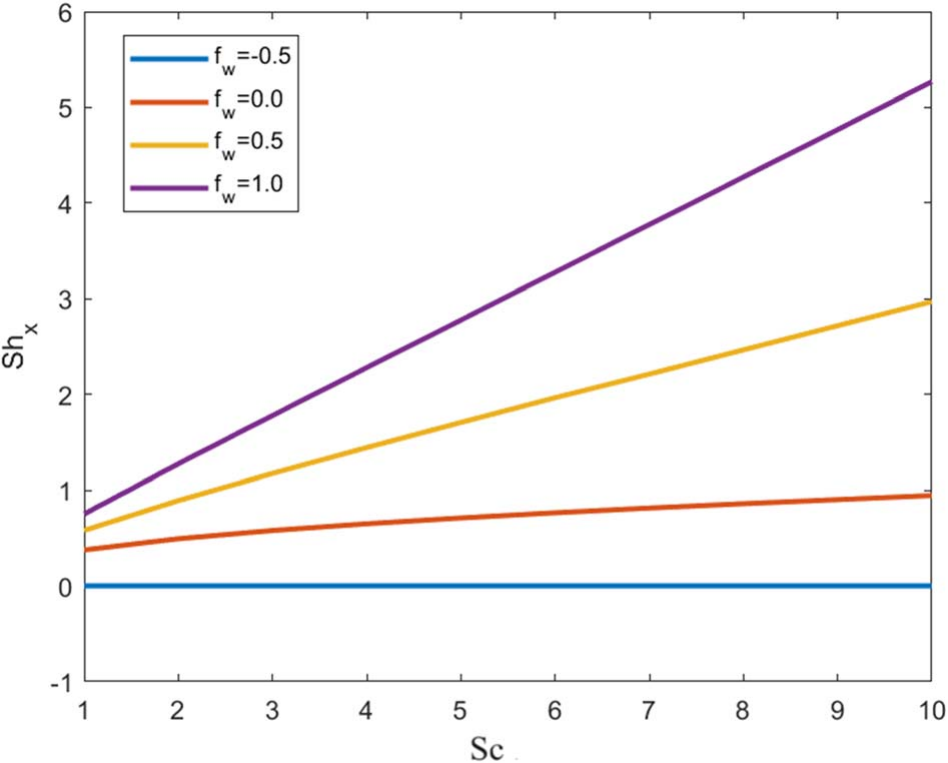
Sherwood number profile for the swelling value of the suction effect against Sc.

Fig. 1–9 display the temperature profile, concentration profile and stream line results for various values of *β*, *λ*, *f*_w_, and Sc. Fig. 1 illustrates the streamline under conditions without suction parameters, while Fig. 2 showcases the same streamline with the inclusion of suction. Lastly, Fig. 3 provides insight into the fluid flow behavior in the absence of momentum slip. In fluid flow, the no-slip boundary condition is a fundamental concept that describes the behavior of fluid molecules at a solid boundary or surface. When suction is equal to zero on a flat plate (meaning that there is no mass being extracted from the fluid at the plate's surface), the streamlines of the flow play a significant role in describing the behavior of the fluid. With suction equal to zero, there is no net mass transfer between the fluid and the flat plate's surface. This implies that the fluid remains in contact with the plate, and there is no removal or injection of fluid material at the boundary. The absence of suction means that the fluid will closely adhere to the plate's surface according to the no-slip boundary conditions. These conditions dictate zero fluid velocity at the plate's surface. As a result, the fluid follows the contours of the flat plate, and streamlines are shaped accordingly. A boundary layer forms near the plate when a fluid flows on a solid surface with no-slip conditions. This boundary layer is a region where the velocity of the fluid changes significantly from zero at the plate's surface to the free-stream velocity away from the plate. The streamlines within this boundary layer depict the gradual increase in velocity from zero at the wall to the free-stream velocity. As one moves away from the flat plate (vertically upward, for example), the streamlines start to resemble those of the free-stream flow. The streamlines farther away from the plate will have a nearly constant velocity, and the presence of the boundary layer will lessen their influence.

### 4.2 Temperature profile

• Observing the behavior of temperature profiles, it becomes evident that as the thermal slip conditions increase incrementally, there is a noticeable reduction in temperature. Thermal slip, in this context, means the heat exchange between the fluid and the thermal flow at the wall boundary, resulting in heat loss. Fig. 4 elucidates the impact of increasing thermal slip parameters on thermal distribution. It is apparent that thermal diffusion diminishes as the thermal slip conditions grow. This phenomenon occurs due to the amplification of the thermal gradient. Thermal slip conditions pertain to how temperature behaves at the fluid and solid interface. In the absence of thermal slip (commonly referred to as no-slip conditions), the temperature at the solid surface is typically assumed to be equal to the solid surface temperature. However, when thermal slip is present, the fluid's temperature at the solid boundary may differ from the solid surface temperature. Positive thermal slip allows for the adjustment of energy transfer between the solid and the fluid, either enhancing or diminishing it, depending on the temperature gradient. Analogous to the velocity boundary layer in fluid flow, a temperature boundary layer forms near the solid surface. Within this layer, the fluid's temperature undergoes a rapid transition from the wall temperature (usually constant) to the bulk temperature of the fluid. The thickness of this thermal boundary layer hinges on the thermal properties of the fluid and the intensity of the slip conditions. As thermal slip conditions intensify, the slip velocity of fluid particles at the solid–fluid interface increases. This slip velocity characterizes the relative motion between the fluid and the solid surface, which can significantly influence heat transfer by altering energy transport from the solid surface into the fluid. Increasing slip velocity can lead to a reduction in thermal conduction at the solid–fluid interface.

• In the conventional no-slip scenario, fluid particles near the wall have zero velocity, and heat transfer primarily occurs through conduction. Nevertheless, thermal slip introduces relative motion, which can impede this conduction. Conversely, the slip conditions can enhance convective heat transfer within the thermal boundary layer. The relative motion between the fluid and the solid surface promotes increased heat transport through convection. As the slip conditions strengthen, this convective heat transfer may surpass thermal conduction. Consequently, these mechanisms contribute to deviations in the temperature outline within the thermal boundary layer compared to the expected profile under no-slip conditions. The precise temperature distribution relies on the specific slip conditions, fluid properties, and flow parameters at play.

• The suction parameter (by considering *f*_w_ = 0, 0.5, 1.0, 1.5) influence on the temperature outline is presented in Fig. 5; *f*_w_ leads to a fall-off in the temperature outline. Suction can also induce mixing of fluids at different temperatures, which can lead to complex temperature profiles and gradients near the boundary. The temperature profile on a horizontal plate is closely related to the thickness of the thermal boundary layer. The thermal boundary layer is the region of the fluid where the temperature changes significantly as it adjusts from the wall temperature (usually a constant) to the bulk temperature of the fluid. The thinner velocity boundary layer with suction also implies a thinner thermal boundary layer. Suction reduces the thickness of the thermal boundary layer, which means less fluid is available to conduct heat from the solid surface. This can result in a decrease in thermal conduction through the fluid near the plate. Despite the reduced thermal conduction, suction can enhance convective heat transfer. The thinner boundary layer and higher fluid velocity can lead to more efficient convective heat transport. This effect can dominate over the reduction in thermal conduction, mainly when the suction is strong. The combined effect of reduced thermal conduction and enhanced convective energy transfer leads to changes in the overall temperature gradient near the flat plate.

• In many cases, the temperature gradient becomes steeper with suction because heat is transported away from the surface more efficiently due to enhanced convection. Depending on the specific suction conditions, suction can also lead to a more uniform temperature profile across the flat plate. This is because it reduces the temperature difference between the surface and the bulk fluid, making the temperature more consistent along the plate's length.

### 4.3 Concentration profile

• Fig. 6 presents the behaviour of the concentration field by considering *λ* while the other parameter values remain fixed. The enhancing tendency of *λ* leads to a decrease in the concentration outline. Velocity slip conditions can lead to enhanced mass transfer near the solid surface. The relative motion between the fluid and the surface can create a stirring or mixing effect as the fluid flows over the plate. This increased mixing can enhance the transport of the solute to and from the surface. Similar to the velocity boundary layer in fluid flow, a concentration boundary layer is near the solid surface. This layer represents the region in which the concentration of the solute changes rapidly from the surface value to the bulk value of the fluid. With velocity slip conditions, the boundary layer thickness can be affected. The thinner the boundary layer, the more rapid the changes in concentration. In addition to the enhanced mixing, the relative motion between the fluid and the surface can increase the effective diffusion of the solute in the boundary layer. This is because the solute molecules experience more frequent collisions and interactions with the fluid due to the slip conditions, which can promote mass transport. The balance between convection and diffusion becomes more pronounced with slip conditions. Convection refers to the transport of solute by the bulk fluid flow, while diffusion is the process by which solute molecules move randomly due to their thermal motion. The presence of slip can alter this balance, potentially favoring convection over diffusion.

• Fig. 7 represents the concentration profile under the influence of Sc. It is spotted that the concentration outline is reducing to increase the value of Sc. When the Shmith number is greater than 1, it means that thermal conduction (heat transfer) is more efficient than molecular diffusion (mass transfer). In this scenario, heat is transported more rapidly than mass, leading to a situation where the temperature field changes more rapidly than the concentration field. With Sc > 1, the concentration profile tends to be more diffuse compared to the temperature profile. This means that the concentration of a solute or component in the fluid diffuses more slowly than the temperature changes. As a result, one may observe a broader and more gradual transition in concentration.

• The suction parameter (by considering *f*_w_ = 0, 0.5, 1.0, 1.5) influence on the concentration field is presented in Fig. 8. *f*_w_ leads to an increase in the concentration field and fall-off in the solute field. Suction refers to removing fluid at the solid boundary, and it can have several effects on the concentration distribution, such as enhanced mass removal: as the suction effect increases, more fluid or solute is removed from the vicinity of the solid boundary. This means that the concentration of the solute near the boundary reduces more rapidly. The enhanced mass removal leads to a steeper concentration gradient near the solid surface. Thinner boundary layer: the suction effect can thin the boundary layer, which is the region near the solid boundary where concentration gradients are significant. As more fluid is removed, the boundary layer becomes thinner, and the concentration within this region decreases more rapidly. Increased mass transport: with increasing suction, mass transport (*i.e.*, the transfer of the solute) from the bulk fluid to the boundary occurs at a higher rate. This results in more efficient solute removal from the solid surface's vicinity. Reduced concentration levels and higher suction levels can lead to reduced maximum concentration levels near the boundary. This is because the suction effect continuously removes solute from the fluid adjacent to the solid surface, preventing the accumulation of high concentrations. Concentration boundary conditions: depending on the specific problem and boundary conditions, the increased suction effect may alter the concentration boundary conditions at the solid surface. For example, in cases where the boundary conditions are specified as a constant concentration or as a function of time, the suction effect can influence the rate at which these conditions are approached.

### 4.4 Sherwood number profile

**Fig. 10:**
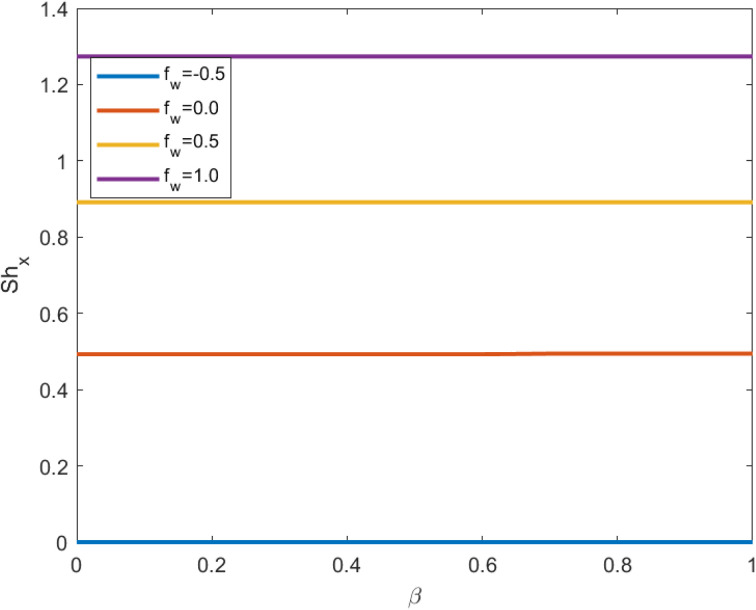
Sherwood number profile for the cumulative value of the suction effect against the thermal slip effect.

Fig. 9 and 10 depict the Sherwood number profiles as the suction effect, thermal slip conditions, and Schmidt number are varied. The underlying physical mechanism driving these variations involves mass transfer, fluid dynamics, and heat transfer within a fluid-solid boundary layer. The suction parameter characterizes the strength of suction applied at the interface between the solid and the fluid. Suction, when applied, draws the fluid closer to the solid surface, consequently modifying the velocity outline near the boundary. Suction diminishes the fluid velocity near the solid surface, which, in turn, impacts the rates of mass and energy transfer. As the suction parameter increases, the near-surface velocity decreases even further. Suction boosts mass transfer by intensifying the concentration gradient near the solid surface.

Consequently, this elevation in the concentration gradient results in a higher Sherwood number, indicating more efficient mass transfer. Suction also exerts an influence on heat transfer. The modified velocity profile and concentration gradient influence the thermal boundary layer, leading to alterations in the temperature distribution near the solid surface. This, in turn, affects the Sherwood number *via* the Schmidt number (Sc). Thermal slip conditions arise when a temperature difference exists between the fluid and the solid surface at the boundary. Thermal slip can modify the temperature gradient in the vicinity of the solid surface. As the thermal slip parameter increases, the temperature difference between the fluid and the solid surface becomes more pronounced. The Schmidt number (Sc) establishes a relationship between momentum diffusivity (kinematic viscosity) and mass diffusivity (the diffusivity of the solute in the fluid), playing a pivotal role in mass transfer. Temperature changes, induced by thermal slip, can have implications for Sc. The interplay between heat and mass transfer is regulated by Sc. Variations in Sc, driven by thermal slip, influence the concentration profile and, consequently, the Sherwood number. Sc is a dimensionless parameter that characterizes the relative significance of momentum transfer (viscous effects) and mass transfer (diffusion effects) within a fluid. Higher Sc values imply slower mass transfer compared to momentum transfer, and they typically lead to reduced Sherwood numbers since they indicate less effective mass transfer.

### 4.5 Strengths of this study

Incorporating Maxwell fluids into the study allows for more realistic modeling of complex fluids that exhibit both viscous and elastic behavior. These fluids are encountered in various practical applications, including polymer processing, biological flows, and certain types of lubricants. This research can provide valuable insights into the interplay between viscoelasticity (represented by the Maxwell fluid model) and slip effects on the fluid flow and heat transfer characteristics. Understanding how these factors interact can lead to improved designs and control strategies in applications involving such fluids. The study of Maxwell fluid flow with slip conditions and suction is inherently interdisciplinary, applying principles from fluid mechanics, rheology, heat transfer, and materials science. It can bridge gaps between these fields and promote collaboration. This research has applications in microfluidics and nanofluidics, where slip conditions become more prominent due to the small length scales involved. Understanding the behavior of complex fluids in these contexts is crucial for the design of microfluidic devices. Combining Maxwell fluids with slip and suction conditions can lead to the discovery of novel phenomena or unexpected behavior in fluid dynamics and heat transfer, which may have practical implications or stimulate further research.

### 4.6 Limitations of the current study

The combined study of Maxwell fluid flow with velocity slip, thermal slip, and suction effects is theoretically and computationally complex. The mathematical equations governing such systems can be challenging to solve analytically, and numerical simulations may require significant computational resources. Analytical solutions for such complex systems may often need to be more readily available, and researchers may rely heavily on numerical methods. This can make obtaining closed-form expressions for key flow and energy transfer parameters difficult. The behavior of Maxwell fluids and the effects of slip and suction can be highly sensitive to parameters such as relaxation time, slip lengths, and suction rate. Determining the most influential parameters and their values can be non-trivial. Conducting experiments to validate theoretical and numerical findings in such complex systems can be challenging and expensive, especially at the microscale or nanoscale. Results obtained for specific combinations of parameters and boundary conditions may not generalize easily to other systems, making it necessary to carefully define relevant parameter regimes.

## 5 Conclusions

This study examines the numerical representation of fluid flow following the Maxwell model in a double-diffusive boundary layer on a horizontal plate. The investigation incorporates slip conditions, encompassing momentum slip, thermal slip, and suction parameters. Moreover, the study incorporates the effects of radiation, energy generation, and mass transfer. The governing PDEs (pertaining to momentum, continuity, energy transport, and mass transport) are transformed into ODEs by utilising appropriate similarity transformations. To solve this system of equations in conjunction with suitable boundary conditions, we employ the built-in “bvp4c” software in MATLAB. A comprehensive agreement between the numerical technique and previously published findings demonstrates its efficacy. The outcomes are presented through graphical representations and tables, showcasing various parameters such as momentum slip, temperature slip, local Nusselt number, Sherwood number, and suction parameter. This investigation allows us to draw the following conclusions:

• A streamline profile is provided for three different scenarios: one with a no-slip effect, another with a slip effect, and the third depicting a zero-velocity profile.

• The temperature profile diminishes as the thermal slip and suction effects increase.

• The concentration profile decreases as the velocity slip, Schmidt number, and suction effect increase.

• The Sherwood number profile increases with growing values of the suction effect in contrast to the Schmidt number and thermal slip conditions.

## Nomenclature

*C*_f_
: Skin friction coefficient
Rd: Radiation parameter
σ*: Stefan–Boltzmann constant
*C*
: Concentration of the fluid
*v*_w_
: Dimensional suction effect
u* and v*: Velocity of the fluid
*C*_p_
: Specific heat
De: Deborah number
*k*
: Thermal conductivity
*f*
: Dimensionless stream function
*θ*
: Non-dimensional temperature
*σ*
: Fluid's electrical conductivity
*f*_w_
: Dimensionless suction parameter
(*ρc*_p_): Heat capacity
Ec: Eckart number
*μ*
: Dynamic viscosity
*η*
: Similarity variable
*ρ*
: The fluid density
Nu_*x*_: Nusselt number
*Q*_1_
: Heat generation
*x*
: Streamwise coordinate
*q*_r_
: Radiative heat flux
Pr: Prandtl number
*C*_∞_
: Concentration on the free stream
*β*_1_
: Dimensional thermal slip condition
*T*
: Temperature of the fluid
*λ*_1_
: Dimensional velocity slip condition
*ν*
: Kinematic viscosity
*T*_∞_
: Free stream temperature
*ϕ*
: Dimensionless concentration
*T*_w_
: Temperature on the plate
*D*_B_
: Mass diffusion
*y*
: Coordinate normal to the plate
*λ*_2_
: Relaxation time
*C*_w_
: Concentration on the plate
*U*_∞_
: Free stream velocity
Sc: Schmith number
*λ*
: Dimensionless velocity slip condition
*β*
: Dimensionless temperature slip condition
*f*_w_
: Dimensionless suction effect
Sh_*x*_: Sherwood number
Re: Reynold number

## Conflicts of interest

There are no conflicts to declare.

## Supplementary Material
